# The Impact of Resilience on Workplace Violence Experienced by Mental Health Nurses: A Cross-Sectional Survey

**DOI:** 10.1155/2024/4449445

**Published:** 2024-04-02

**Authors:** Atallah Alenezi

**Affiliations:** Department of Nursing, College of Applied Medical Sciences, Shaqra University, Shaqra, Saudi Arabia

## Abstract

**Background:**

Violence at work against healthcare professionals is a frequent and pervasive problem. There are growing data that indicate nurses are especially susceptible to experiencing violent acts at work. Resilience helps strengthen nursing competency in the proper management of stressful circumstances, like being exposed to workplace violence.

**Aim:**

The aim of the study is to assess the impact of resilience on workplace violence among mental health nurses.

**Method:**

A cross-sectional research design was used to conduct this study with a convenience sample of 361 nurses recruited from a governmental psychiatric hospital in Saudi Arabia. *Tools*. Data were collected using two tools: the first tool was the workplace violence questionnaire, which collected nurses' demographic, job, and workplace violence data, and the second was the resilience at work scale to assess nurses' resilience.

**Results:**

This survey found that 70.4% of nurses experienced workplace violence in the last year, and fewer than half were resilient at work. Close to one-third (33.5%) of nurses were terrified and confused after workplace violence. The most violent repercussions were psychological (46.8%): dread, tension, and worry. Additionally, 48.8% of nurses exhibited high work resilience.

**Conclusion:**

A considerable percentage of mental health nurses encountered instances of violence during the provision of care inside mental health hospital settings. The variables of nationality (non-Saudi nurses), rotating work shift, educational levels, and exhibiting a lower level of resilience were found to have a statistically significant correlation with instances of workplace violence. *Recommendation*. Training programs and educational initiatives should be developed and implemented to equip nurses with the necessary knowledge and skills to effectively respond to and prevent workplace violence incidents. By providing comprehensive training, healthcare organizations can empower nurses to address and mitigate workplace violence, ultimately creating a safer and more supportive work environment.

## 1. Introduction

Workplace violence poses a significant health concern for healthcare workers globally, including mental health nurses, who are particularly vulnerable to such incidents [1]. Workplace violence is defined as any act of physical abuse, intimidation, harassment, or threatening behavior occurring at the workplace; it encompasses a range of behaviors from verbal abuse to homicide [2]. Among the perpetrators, workplace violence was done by patients' relatives, patients, staff, and management [3].

Healthcare and social service personnel, including nurses, are particularly vulnerable to client violence, which accounts for 30% of all workplace homicides [2]. The prevalence of workplace violence within healthcare settings is not limited to a single profession but extends across various sectors within the healthcare industry [4]. For instance, studies have reported a high prevalence of workplace violence among healthcare workers, including nurses, physicians, support staff, and paramedics. In the district of Peshawar, Pakistan, the estimated prevalence of workplace violence was found to be 51.0% [5]. Similarly, in northwest Ethiopia, more than half (58.2%) of healthcare workers, including nurses/midwives, physicians, general practitioners, and pharmacists/laboratory staff, reported experiencing at least one manifestation of workplace violence (physical, verbal, or sexual) in the past 12 months [6]. Recent research has also indicated a high prevalence of workplace violence among registered nurses, ranging from 70.0% to 80.0% [7]. Furthermore, a study conducted in upper Northern Thailand found that the prevalence of physical workplace violence among nurses in the preceding 12 months was 12.1%, while the prevalence of psychological violence included verbal abuse (50.3%), bullying/mobbing (10.3%), and sexual harassment (1.6%) [8]. This elevated risk is attributed to the inherent nature of their roles and frequent interactions with patients, families, and visitors [9]. Workplace violence can occur across various healthcare settings, including hospitals, nursing homes, primary health care setting, and psychiatric facilities [4, 5].

Numerous factors contribute to the risk of workplace violence, including working closely with individuals with a history of violence, social and occupational pressures, organizational policies, employees' psychological attributes, and personal characteristics [10–12]. Nurses, in particular, are at risk due to their close proximity to patients and their families, who may exhibit aggressive behaviors due to illness, injury, or stress [13]. Najafi et al. [14] identified five categories of predisposing factors associated with workplace violence. These factors include unmet expectations of patients/relatives, inefficient organizational management, inappropriate professional communication, factors related nurses as poor stress management and lack of clinical competence among nurses, and factors related to patients, patients' relatives, and colleagues such as mistrust of nurses' job performance and a negative public image of the nursing profession.

Workplace violence has detrimental effects on nurses, leading to decreased job satisfaction, increased absenteeism, and poor service quality [15]. In acute psychiatric settings, nurses face heightened risks of aggression from service users, necessitating the adoption of effective coping strategies and resilience-building initiatives [16].

Resilience, characterized by self-awareness, faith, hope, insight, and self-care, plays a crucial role in mitigating workplace violence among nurses [17]. Developing personal resilience helps nurses to manage stress, preserve job satisfaction, and enhance job performance. Resilience interventions have shown promising results in reducing workplace violence and strengthening nurses' ability to cope with challenging situations [18].

There are several methods that can help nurses build their resilience. Providing comprehensive education and training on stress management techniques, self-care practices, and coping strategies is crucial [19]. Workshops, seminars, and ongoing professional development programs can equip nurses with the necessary tools to navigate difficult situations successfully. Additionally, establishing peer support networks and mentorship programs allows nurses to connect with and learn from their colleagues, fostering resilience through shared experiences and emotional support [20]. Developing emotional intelligence skills, such as self-awareness, empathy, and effective communication, further aids nurses in managing their emotions and responding empathetically to patients and colleagues, thereby enhancing their resilience [21]. Moreover, introducing mindfulness and relaxation practices, such as meditation, deep breathing exercises, and yoga, can also contribute to resilience by promoting self-awareness, stress reduction, and emotional well-being [22]. Finally, promoting a healthy work-life balance through policies that prioritize adequate rest, time off, and scheduling flexibility is vital for nurses' resilience [23]. By recognizing the value of building resilience in nurses and allocating resources to support these concrete methods, healthcare organizations and leaders can create a more supportive and sustainable work environment that prioritizes the well-being and resilience of nurses.

To my knowledge, the existing research on workplace violence in healthcare has primarily focused on its prevalence, risk factors, and consequences for healthcare professionals in general. However, limited attention has been given to the role of resilience as a potential protective factor when mental health nurses in Saudi Arabia face workplace violence. While resilience has been recognized as a valuable resource for coping with occupational stress and adversity, its specific impact on workplace violence experienced by mental health nurses remains underexplored. Thus, the research gap lies in the need to investigate the relationship between resilience and workplace violence in the context of mental health nursing, providing a deeper understanding of how resilience can contribute to preventing or mitigating workplace violence incidents. This study aims to address this research gap by examining the impact of resilience on workplace violence experienced by mental health nurses.

### 1.1. Research Questions

1. What are the levels of workplace violence and resilience among mental health nurses?
2. Is there a relationship between resilience and workplace violence among mental health nurses?

## 2. Methods

### 2.1. Research Design and Setting

This study utilized a cross-sectional design to quantitatively assess the prevalence of an outcome (workplace violence) and associate various factors (resilience) at a single point in time. This study was conducted at a large, 530-bed inpatient psychiatric facility in Riyadh, Saudi Arabia. This government-run mental health center serves both adult and child patients from the local community presenting with a range of acute and chronic psychiatric conditions.

### 2.2. Participants

The present study utilized a convenience sampling method, consisting of 361 mental health nurses. The nurses included in the study were of both genders, had a minimum of one year of professional experience, showed a willingness to actively engage in the research, currently employed, and actively providing direct patient care. On the other hand, nurses with less than one year of clinical experience, part-time nurses, those not currently employed at the mental health center, and nurses occupying administrative or nonpatient care roles were excluded from the study.

### 2.3. Sample Size

The sample size was calculated using the formula *n*=(*Z*2 *∗* *σ*2)(*p*)(*q*)/*d*2, where *n* indicates the required sample size, *z* is the standardized normal deviation corresponding to *α* = 0.05 and 95% confidence level (*z* = 1.96, for two-tailed), *p* indicates the expected prevalence based on the previous study [24] (*p*=90.3%), *q* indicates 1 − *p*, and *d* indicates the acceptable margin of error for the mean (*d* = 5%). Consequently, a minimum sample size of 135 was initially determined, with consideration for an anticipated 10% attrition rate. Subsequently, the minimum required sample size should be 149 nurses. The researcher distributed a total of 406 questionnaires in order to ensure an adequate representation of the population and maintain statistical power. Out of the 406 questionnaires, 45 were found to be incomplete, leaving a total of 361 completed questionnaires for analysis.

## 3. Tools for Data Collection

### 3.1. Tool I: Resilience at Work Scale

This study utilized the resilience at work scale, which was adapted from the work of Winwood et al. [25], to assess resilience among mental health nurses. It comprises 17 items that are categorized into six dimensions: living authentically (3 items), finding your calling (3 items), maintaining perspective (2 items), managing stress (4 items), building social connections (3 items), and staying healthy (2 items). The Likert scale score for the nurses' responses was reduced from 7 to 5 points based on the expert opinions and recommendations. The scores on the scale range from 1 (strongly disagree) to 5 (strongly agree). For every negative statement, the score will be reversed. The overall score ranges from 17 to 85. Low resilience is shown by a score between 17 and 39, moderate resilience is shown by a score between 40 and 62, and high resilience is revealed by a score between 63 and 85.

### 3.2. Tool II: Workplace Violence Questionnaire

This questionnaire was developed by the researcher after reviewing recent literature [26–28]. It consisted of three components. The initial section focused on the demographic attributes of the participants, including variables such as age, gender, marital status, nationality, and experience. The subsequent phase of the study examined workplace data, working shifts, and the presence of workplace violence prevention techniques, including training programs, policies, and safety practices. The third part presents information pertaining to the empirical exposure of individuals to occurrences of workplace violence. The study included several aspects of violence, including its nature, origin, duration of occurrence, individuals responsible for its perpetration, responses to occurrences of violence, and suggestions provided by nurses to mitigate the exposure to such acts of violence.

Seven mental health and community care specialists examined the two instruments for content validity to assess their relevance, clarity, application, question phrasing, and interview duration. Following input, changes were made. Cronbach's alpha assessed tool dependability. The resilience scale's reliability coefficient was 0.82, which indicates strong reliability. A pilot study with 30 nurses tested tool feasibility and clarity. Therefore, the appropriate changes were made. The pilot study nurses were omitted from the main sample of the study.

### 3.3. Ethical Consideration and Data Collection

The researcher gained approval from the Committee of Research Ethics at Shaqra University (ERC-SU-S-202400005). A formal correspondence was sent to the research institution to request authorization for the collection of essential data. Following a comprehensive explanation of the study's objective, authorization was obtained from the directors and departmental heads of the designated hospital to proceed with participant recruiting. Following a comprehensive elucidation of the study's objective, the researcher diligently collected signed informed permission from the participating nurses prior to the commencement of data collection. All individuals who took part in the study were provided with a guarantee of both anonymity and confidentiality, which extended beyond the duration of the research. To ensure participant anonymity, stringent, each participant was assigned a unique code that was used to identify their responses and data. This code was not linked to any personally identifiable information, ensuring the confidentiality of their identity. The participants were not provided with cash or monetary incentives for their participation in the study. However, the researcher expressed his appreciation for their involvement through acknowledging their contribution to the research and emphasizing the importance of their participation in generating valuable insights for the field. The importance of participants' voluntary engagement and their entitlement to resign from the research at any given point were also underscored. If a participant chose to withdraw, their data would be immediately removed from the dataset using their unique identifier assigned to the participant. Data collection began in January 2023 and finished in April 2023. After briefly outlining the study's goal and the requirement to gather data, the questionnaire was delivered individually in the study setting.

### 3.4. Data Analysis

The data that were gathered were systematically arranged, compiled into tables, and subjected to statistical analysis using IBM SPSS, version 23.0. The assumption of normality was acceptable, with categorical variables being expressed in terms of frequency and percentage. The mean and standard deviation were used to represent continuous variables. Binary logistic regression was chosen as the primary statistical analysis technique for predictive modeling with a binary outcome variable (workplace violence coded as 0 or 1). Demographic variables were entered into block 1 to control their potential confounding effects and resilience entered into block 2 in the regression model. A *p* value less than 0.05 was deemed to be statistically significant.

## 4. Results

### 4.1. Distribution of Nurses According to Their Demographic Characteristics and Their Relationships with Exposure to Workplace Violence

The majority of the sample consisted of young people, with an average age of 30.31 years. The majority of respondents were male (76.5%), married (79.2%), and of Saudi nationality (71.5%). 52.1% of participants possessed a bachelor's degree in nursing. The mean clinical nursing experience in the sample was 9.97 years. The prevailing work patterns identified were regular shifts (63.4%) as opposed to rotational shifts (36.6%) and working nonmorning shifts (53.2%) (Table 1).

### 4.2. Distribution of Nurses Based on Their Exposure to Workplace Violence

A significant proportion (70.4%) of the nurses experienced workplace violence at least once within the past year, mainly verbal, as reported by the majority (67.3%) of them, followed by physical and sexual violence (15.0%, 9.1%, respectively). Moreover, more than one-third (35.2%) of them were exposed to workplace violence three times or more. More than half (59.0%) of participants were exposed to violence in the morning, mainly by patients (45.4%), and the majority of them were exposed to violence by adult (53.2%) and male perpetrators (34.3%). Nearly one-third (33.5%) of the nurses experienced fear and confusion as immediate reactions to workplace violence. Furthermore, the predominant outcomes of violent incidents were psychological effects, specifically manifesting as fear, stress, and anxiety, accounting for 46.8% of cases. A majority of the nurses, specifically 65.4%, reported occurrences of violent situations (Table 2).

### 4.3. Suggestions from Nurses for the Prevention and Management of Workplace Violence

**Figure 1:**
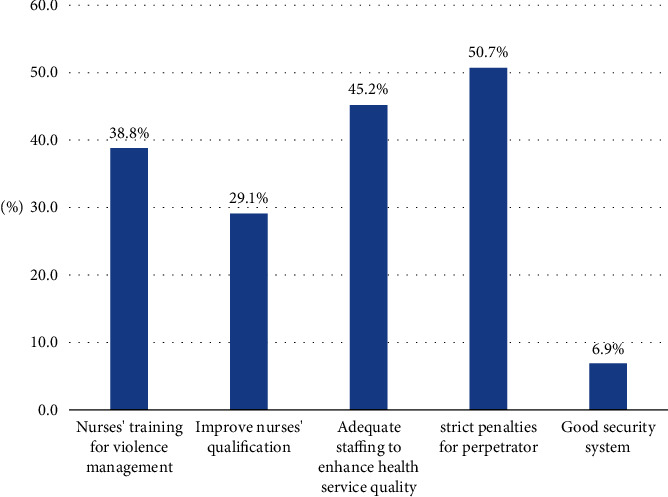
Suggestions from nurses for the prevention and management of workplace violence. Respondents were allowed to select multiple options.

Strict penalties for violence perpetrators were the most frequent suggestion by more than half (50.7%) of the nurses, followed by adequate staffing to enhance health service quality (45.2%), attending training programs focused on violence prevention and management (38.8%). Additionally, 29.1% of participants indicated prioritizing the enhancement of nurses' qualifications. On the other hand, a minority (6.9%) of the nurses suggested the presence of an effective security system (Figure 1).

### 4.4. Nurses' Levels of Resilience at the Workplace

Most of the nurses had high levels of resilience in relation to the “living authentically” and “building social connections” domains (87.0% and 82.0%, respectively). Over three quarters of the participants, specifically 76.2 percent, exhibited a high level of resilience in the “finding your calling” domain. In contrast, less than half of the participants demonstrated a high level of resilience in the “staying healthy” and “managing stress” domains, with percentages of 47.9% and 46.8%, respectively.

**Figure 2:**
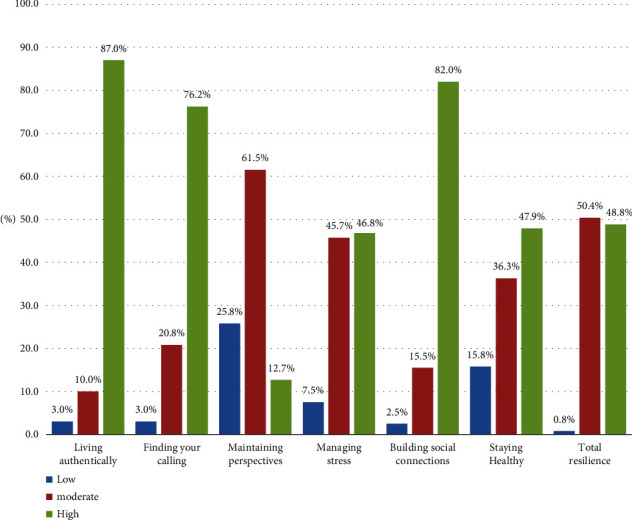
Nurses' levels of resilience at workplace.

Furthermore, slightly less than half (48.8%) of the studied nurses had a high total level of resilience at work (Figure 2).

### 4.5. Predictors of the Nurses' Exposure to Workplace Violence

The association between exposure to workplace violence and the characteristics of the participants was explored using binary logistic regression analysis (enter method), with exposure to workplace violence as the dependent variable. The overall model was statistically significant, *χ*^2^(11) = 53.95, *p* < 0.001, indicating the predictors as a set reliably distinguished between exposure groups. The Cox & Snell R square was 0.139, suggesting that the predictors collectively explain about 13.9% of the variation in the nurses' exposure to workplace violence. Only four variables were found to be predictors of exposure to workplace violence such as nationality, education, work rotation, and resilience at work. The ORs (95% CI) for the nationality (reference: Saudi) were 0.55 (0.32–0.95). The ORs (95% CI) for secondary school education, technical institute, and bachelor's degree were 3.79 (1.30–11.02), 3.94 (1.08–14.40), and 2.92 (1.03–8.21), respectively. The ORs (95% CI) for work rotation types and resilience were 2.41 (1.15–5.05) and 0.92 (0.89–0.95), respectively (Table 3).

## 5. Discussion

The present study had two objectives: firstly, to evaluate the prevalence of workplace violence and resilience among mental health nurses; secondly, to delve into the intricate relationship between resilience and the perception of workplace violence among mental health nurses.

The findings of the current survey indicate that more than two thirds (70.4%) of the nurses reported instances of workplace violence over the last year. This finding comes in line with the results of Basfr et al. [24] who found that the prevalence of workplace violence among mental health nurses was 90.3%, alarming that nurses experience such a high level of violence. Furthermore, the finding of the present study noted that verbal violence was the most common type of violence experienced by most nurses, followed by physical violence. Similar findings were reported by Sun et al. [29] and Ferri et al. [30] who found that the most frequent type of violence was verbal abuse. Moreover, the current study found that the less common type of workplace violence was sexual violence (9.1%). This might be attributed to the fact that sexual violence and assault are deplorable actions across different communities and go against both the law and religious principles. This is supported by the results of Harthi et al. [31] who noticed that the rate of sexual harassment was lower than those reported in other countries and justified this lower prevalence of sexual assaults due to the nature of the Saudi community and its compliance with religious values and rules. Additionally, numerous effects of workplace violence have been documented in several studies, and these effects significantly affect employees' physical and mental health as well as their productivity and level of customer service [32, 33].

The study findings reported less than half of nurses (46.8%) indicated that they experience psychological effects resulting from their exposure to workplace violence. Additionally, a smaller proportion (17.7%) reported changes in work performance, including absences and decreased productivity, which can significantly affect the quality of care delivered. These findings disagreed with Small et al. [34] who reported that most of the participants were under-reporting workplace violence incidents, mainly because of their unawareness of the availability of such policies and not receiving training in the workplace. From the results of the present study, it could be observed that around half of the studied nurses were exposed to workplace violence three and more attacks in the last year. The study's findings supported with Clari et al. [35] and Sisawo et al. [36] who reported that most health care workers experienced three and more violent events. Like many studies, the patients and their relatives were frequently reported as the main perpetrator of violence toward nurses [3]. This might be accounted for by the fact that nurses are frequently the first-person that patients and their families meet and have more direct interactions with them. They are frequently held responsible for late or subpar health services. As a result, they take the brunt of patients' unruly behavior, their relatives' dissatisfaction with the care they receive, and/or the confusing hospital rules that outsiders are expected to follow. In a sense, nurses end up being the scapegoat. Additionally, nurses who are understaffed and compelled to hurry care increase patient and family dissatisfaction with the care they receive, raising the risk of violence in the workplace [37].

Unfortunately, the current study noted that less than half of the studied nurses reported occurrence of violent acts from their coworkers such as physicians, nurses, and supervisors. This is not what one would expect, as a safe environment should be provided for both patients and the coworkers themselves in a health care setting that is free from threats of violence. In the same context, the result of Ope-babadele and Ilesanmi [38] found that physicians and supervisors were among the sources of workplace violence against nurses.

Furthermore, the results from the current study highlight that nurses who were working rotating shifts and evening and night shifts reported more exposure to violent attacks. This finding aligns with the research conducted by Sun [29], which revealed a correlation between heightened risks of work-related violence and nurses who were assigned to evening, night shifts, and rotating day schedules. This may be due to inadequate security and having fewer nurses' numbers in comparison to the morning shift creating an environment that is conducive to violence. For instance, the study of Chaiwuth et al. [8] found that risk factors for verbal abuse included being a registered nurse with direct nursing care responsibilities; workplaces without adequate security; and having workplace violence concerns. Moreover, the study of Basfr et al. [24] found that the time of violence, source of violence, patient dissatisfaction with medical care, and lack of organizational support for nurses were significantly associated with the occurrence of workplace violence in psychiatric units.

The current study found that the majority of the nurses exhibited either high or moderate levels of resilience, indicating their ability to effectively cope with and adapt to workplace challenges. These findings align with the results of an integrative review study conducted by Bui et al. [1], which examined the resilience levels of mental health nurses. The review study found that mental health nurse resilience tends to be moderate to high. Also, a literature review study of foster et al. [39] found that resilience in mental health nursing is most often moderate, with positive correlations with hardiness, self-esteem, life and job satisfaction, and negative correlations with depression and burnout. However, the study of Zheng et al. [40] conducted at the Institute of Mental Health in Singapore demonstrated that psychiatric nurses have a moderately low level of resilience. Delgado et al. [41] reported that mental health nurses in Australia had high level of resilience.

From the results of the present study, it could be observed that nurses with high level of resilience are less exposed to workplace violence. This could be attributed to the fact that resilience is seen as a person's positive and constructive responses to stressful situations to promote healthy behaviors. These findings supported by the study Alonazi et al. [42] found that higher resilience levels were associated with higher levels of compassion satisfaction and lower levels of secondary traumatic stress among mental health nurses. Furthermore, an integrative review study of Bui et al. [1] demonstrated that resilience was positively associated with psychological well-being, post-traumatic growth, and compassion satisfaction and negatively associated with burnout, mental distress, and emotional labor among mental health nurses. Notably, Yang et al. [43] emphasized the importance of prevention, de-escalation skills, effective coping strategies, and communication skills in reducing workplace violence among mental health nurses. Similarly, Fida et al. [44] conducted a time-lagged study that highlighted the protective role of relational occupational coping self-efficacy in shielding nurses from workplace incivility, burnout, mental health issues, and turnover intentions. Furthermore, Hsieh et al. [45] demonstrated that personal strength, social competence, and a structured approach serve as protective factors against depressive tendencies in emergency department nurses exposed to workplace violence. Building on these findings, Lozano et al. [12] conducted a systematic review, revealing a significant association between workplace violence and burnout symptoms among nurses and physicians. They identified risk factors such as structural and personal factors, while highlighting the role of a quality work environment and effective coping strategies as protective factors. In the context of healthcare, Morphet et al. [46] conducted a systematic review that underscored the effectiveness of consumer risk assessment, staff education, and aggression management teams in reducing workplace violence. However, their findings indicated no evidence to support the efficacy of zero tolerance policies, incident reporting, and duress alarms. Furthermore, d'Ettorre et al. [19] emphasized the importance of prioritizing training, improved communication skills, accurate reporting, and optimized workplace design in minimizing stressful conditions in waiting rooms to effectively manage workplace violence against healthcare workers in emergency departments. Additionally, Hsieh et al. [47] identified higher family support as a key protective factor against the development of depressive symptoms among assaulted psychiatric ward nurses.

### 5.1. Limitations of the Study

This study had several limitations. Firstly, the findings may lack generalizability to broader populations of healthcare workers or other settings due to the homogeneity of the sample. The exclusive focus on mental health nurses limits the extrapolation of results to healthcare professionals in different specialties or roles who may face distinct risk factors for workplace violence. Moreover, employing convenience sampling introduces potential selection bias, as participants may not be representative of the entire population of interest, leading to skewed findings and decreased external validity. Additionally, the use of a cross-sectional design restricts the ability to establish causal relationships between variables, as it provides only a snapshot of data at a single point in time, precluding longitudinal assessment of trends and changes over time. Therefore, future research endeavors should strive for greater diversity in samples, employ more rigorous sampling methodologies, and consider longitudinal or mixed-methods approaches to enhance the robustness and generalizability of findings regarding workplace violence in healthcare settings.

### 5.2. Implications for Practice

Based on the research findings, it is evident that workplace violence is a significant concern among mental health nurses, with a majority of them reporting incidents of violence within the past year. The study findings also indicate that the level of resilience among these nurses ranged from moderate to high. This suggests that while some nurses demonstrated moderate resilience levels, others exhibited higher levels of resilience. The varying range of resilience levels highlights the diversity among nurses in their ability to adapt and cope with the challenges they face in their roles. It is also important to note that resilience plays a crucial role in protecting nurses from the negative effects of workplace stressors, including violence. The study emphasizes the need for healthcare administrators and policymakers to address the issue of workplace violence and prioritize interventions aimed at enhancing resilience among mental health nurses. By investing in initiatives that promote resilience, healthcare organizations can better support nurses in coping with workplace challenges and mitigating the psychological consequences of violence.

## 6. Conclusion

Based on the results of the present research, it can be inferred that most of the nurses surveyed had occurrences of workplace violence over the last year. This observation is particularly concerning as it reflects the challenges and risks faced by healthcare professionals in their daily work environment. Furthermore, fewer than half of the participants exhibited a high degree of resilience, while the remaining individuals demonstrated a moderate level of resilience. Resilience plays a key role in protecting the negative effects of workplace stressors, including exposure to violence and aggression. The relatively low level of resilience among mental health nurses suggests a potential vulnerability that declares attention from healthcare administrators and policymakers. Furthermore, a noteworthy inverse relationship was seen between the level of workplace violence experienced by nurses and their level of resilience. This finding highlights the importance of fostering resilience among mental health nurses as a protective factor against the negative impact of workplace violence. Nurses with higher levels of resilience may be better equipped to cope with the stressors associated with their profession.

## Figures and Tables

**Table 1:** Distribution of nurses according to their demographic characteristics and their relationships with exposure to workplace violence.

Characteristics	Exposure to workplace violence				Total (*N* = 361)	
	No (*N* = 107)		Yes (*N* = 254)			
	*N*	%	*N*	%	*N*	%
Age (years)						
20–30	55	28.1	141	71.9	196	71.9
31–40	36	30.8	81	69.2	117	69.2
>40	16	33.3	32	66.7	48	66.7
Mean ± SD	31.07 ± 8.55		29.99 ± 7.82		30.31 ± 8.05	
Gender						
Male	88	31.9	188	68.1	276	76.5
Female	19	22.4	66	77.6	85	23.5
Marital status						
Married	79	27.6	207	72.4	286	79.2
Not married	28	37.3	47	62.7	75	20.8
Nationality						
Saudi	68	26.4	190	73.6	258	71.5
Non-Saudi	39	37.9	64	62.1	103	28.5
Educational qualifications						
Secondary school of nursing	34	28.6	85	71.4	119	33.0
Technical institute of nursing	8	23.5	26	76.5	34	9.4
Bachelor's degree of nursing	54	28.7	134	71.3	188	52.1
Postgraduate studies	11	55.0	9	45.0	20	5.5
Years of experience						
<5	43	28.5	108	71.5	151	41.8
5	8	16.7	40	83.3	48	13.3
10	14	21.2	52	78.8	66	18.3
≥15	42	43.8	54	56.3	96	26.6
Mean ± SD	10.99 ± 8.45		9.54 ± 7.57		9.97 ± 7.85	
Work rotation type						
Regular shift	80	34.9	149	65.1	229	63.4
Rotating shift	27	20.5	105	79.5	132	36.6
Work shift						
Morning	49	25.5	143	74.5	169	46.8
Nonmorning (evening/night)	58	34.3	111	65.7	192	53.2

**Table 2:** Distribution of nurses according to their experience of workplace violence.

	*N*	%
Workplace violence exposure at least one within the past year		
No	107	29.6
Yes	254	70.4
Type of workplace violence#		
Verbal workplace violence	243	67.3
Physical workplace violence	54	15.0
Sexual workplace violence	33	9.1
Frequency of exposure to workplace violence		
Single occurrence	63	17.5
Dual occurrence	64	17.7
Three or more occurrences	127	35.2
Time of exposure to workplace violence#		
Morning	213	59.0
Evening	52	14.4
Both	45	12.5
Perpetrator of workplace violence#		
Patients/clients	164	45.4
Patients' relatives	119	33.0
Coworkers (supervisors, physicians, and nurses)	115	31.9
Sex of perpetrators#		
Male	124	34.3
Female	53	14.7
Both	77	21.3
Age group of perpetrators#		
Adult	192	53.2
Elderly	93	25.8
Immediate response to violence#		
No response	66	18.3
Fear/confusion	121	33.5
Notify manager	65	18.0
Defend self	65	18.0
Call for help	23	6.4
Consequences of workplace violence#		
Psychological consequence	169	46.8
Physical consequence	3	0.8
Work outcomes (absenteeism and lessen performance)	64	17.7
No impact	29	8.0
Reporting of violence incidents		
No	88	34.6
Yes	166	65.4

#Respondents could select more than one answer.

**Table 3:** Binary logistic regression for the predictors of workplace violence experiences among the studied nurses.

	*B*	S.E.	Wald	Sig.	ORs	95% CI for exp (*B*)	
						Lower	Upper
Age	−0.01	0.02	0.14	0.71	0.99	0.95	1.03
Sex	0.44	0.33	1.79	0.18	1.55	0.82	2.93
Marital status	−0.54	0.31	2.97	0.08	0.58	0.32	1.08
Nationality	−0.59	0.28	4.62	0.03	0.55	0.32	0.95
Education							
Secondary school	1.33	0.54	5.98	0.01	3.79	1.30	11.02
Technical institute	1.37	0.66	4.31	0.04	3.94	1.08	14.40
Bachelor's degree	1.07	0.53	4.10	0.04	2.92	1.03	8.21
Postgraduate studies	Reference group						
Experience	−0.04	0.02	3.65	0.06	0.96	0.92	1.00
Type of work rotation	0.88	0.38	5.45	0.02	2.41	1.15	5.05
Shift type	0.20	0.34	0.35	0.55	1.22	0.63	2.39
Resilience at work	−0.08	0.02	22.46	<0.001	0.92	0.89	0.95
Constant	5.25	1.32	15.79	<0.001	189.72		

## Data Availability

The data that support the findings of this study are available on request from the corresponding author. The data are not publicly available due to privacy or ethical restrictions.
